# Identification and validation of an epithelial mesenchymal transition-related gene pairs signature for prediction of overall survival in patients with skin cutaneous melanoma

**DOI:** 10.7717/peerj.12646

**Published:** 2022-01-21

**Authors:** Yucang Shi, Zhanpeng Li, Zhihong Zhou, Simu Liao, Zhiyuan Wu, Jie Li, Jiasheng Yin, Meng Wang, Meilan Weng

**Affiliations:** 1 Department of Plastic Surgery, Affiliated Hospital of Guangdong Medical University, Zhanjiang, China; 2 Graduate School of Guangdong Medical University, Zhanjiang, China; 3 Department of Plastic Surgery, Longhua District People’s Hospital, Shenzhen, China

**Keywords:** Skin cutaneous melanoma, EMT-related gene pairs, Prognosis, Nomogram

## Abstract

**Background:**

We aimed to construct a novel epithelial-mesenchymal transition (EMT)-related gene pairs (ERGPs) signature to predict overall survival (OS) in skin cutaneous melanoma (CM) patients.

**Methods:**

Expression data of the relevant genes, corresponding clinicopathological parameters, and follow-up data were obtained from The Cancer Genome Atlas database. Univariate Cox regression analysis was utilized to identify ERGPs significantly associated with OS, and LASSO analysis was used to identify the genes used for the construction of the ERGPs signature. The optimal cutoff value determined by the receiver operating characteristic curve was used to classify patients into high-risk and low-risk groups. Survival curves were generated using the Kaplan–Meier method, and differences between the two groups were estimated using the log-rank test. The independent external datasets GSE65904 and GSE19234 were used to verify the performance of the ERGPs signature using the area under the curve (AUC) values. In addition, we also integrated clinicopathological parameters and risk scores to develop a nomogram that can individually predict the prognosis of patients with CM.

**Results:**

A total of 104 ERGPs related to OS were obtained, of which 21 ERGPs were selected for the construction of the signature. All CM patients were stratified into high-and low-risk groups based on an optimal risk score cutoff value of 0.281. According to the Kaplan–Meier analysis, the mortality rate in the low-risk group was lower than that in the high-risk group in the TCGA cohort (*P* < 0.001), GSE65904 cohort (*P* = 0.006), and GSE19234 cohort (*P* = 0.002). Multivariate Cox regression analysis indicated that our ERGP signature was an independent risk factor for OS in CM patients in the three cohorts (for TCGA: HR, 2.560; 95% CI [1.907–3.436]; *P* < 0.001; for GSE65904: HR = 2.235, 95% CI [1.492–3.347], *P* < 0.001; for GSE19234: HR = 2.458, 95% CI [1.065–5.669], *P* = 0.035). The AUC value for predicting the 5-year survival rate of patients with CM of our developed model was higher than that of two previously established prognostic signatures. Both the calibration curve and the C-index (0.752, 95% CI [0.678–0.826]) indicated that the developed nomogram was highly accurate. Most importantly, the decision curve analysis results showed that the nomogram had a higher net benefit than that of the American Joint Committee on Cancer stage system.

**Conclusion:**

Our study established an ERGPs signature that could be potentially used in a clinical setting as a genetic biomarker for risk stratification of CM patients. In addition, the ERGPs signature could also predict which CM patients will benefit from PD-1 and PD-L1 inhibitors.

## Introduction

Skin cutaneous melanoma (CM) is a very aggressive malignancy, and its incidence has increased rapidly compared to other cancers in the past few decades (Chernyshov et al., 2019). Unfortunately, the 5-year overall survival (OS) rate of CM patients who have already developed lymph node metastasis is approximately 15% (Bayless & Schneider, 2015). Despite that, patients with early-stage melanoma have a favorable prognosis, as advanced melanoma is the deadliest malignant tumor after leukemia (Rubin & Lawrence, 2009). To date, the tumor-node-metastasis (TNM) stage is still a widely used tool to predict the clinical outcome of patients with CM. However, due to the high heterogeneity of CM, patients in the same TNM stage and receiving the same treatment modality often have different clinical outcomes (Coricovac et al., 2018). Therefore, the TNM staging system cannot effectively aid clinicians to accurately predict individual prognostic outcomes. It is particularly important to develop novel and effective gene signatures to more accurately stratify patients to further improve the prognosis of patients with CM.

Epithelial-mesenchymal transition (EMT) is a complex, multi-step biological process. Through the EMT process, epithelial cells lose their characteristic features and aquire mesenchymal cell phenotypes, including invasiveness and migration (Ye & Weinberg, 2015). EMT has been widely recognized as an important process in tumor invasion, metastasis and in causing drug resistance (Nieto, 2011; Yang & Weinberg, 2008). Numerous studies have shown that EMT is related to the invasion and progression of various cancers (Montemayor-Garcia et al., 2013; Yang, Yuan & Li, 2013; Zhao et al., 2013). Although the biological role of EMT in tumor progression has been studied in-depth, the prognostic value of EMT-related gene sets and the potential functions of these prognostic genes in the progression of EMT remain to be explored. To the best of our knowledge, few studies have constructed signatures based on EMT-related genes to predict the prognosis of patients with CM.

The gradual reduction in the cost of high-throughput sequencing and the rapid development of bioinformatics tools in recent years have greatly contributed to our in-depth understanding of cancer. Nowadays, gene signatures, such as long non-coding RNAs and mRNAs, are becoming increasingly important in the prognosis of CM (Chen et al., 2017; Liu et al., 2020). However, various reasons such as differences in sequencing platforms and inherent biological heterogeneity have hindered the incorporation of these prognostic signatures in a clinical setting (Leek et al., 2010). A new recently established algorithm based on the relative ranking of gene expression levels, effectively solved the problem of expression profile data normalization and scaling, and yielded stable results (Heinäniemi et al., 2013; Li et al., 2017). However, to our knowledge, no study has developed an ERGP signature to predict the prognosis of patients with CM. Thus, the purpose of this study was to use the recently developed algorithm to construct a novel signature based on the EMT-related gene sets to accurately predict the prognosis of CM patients.

## Materials and Methods

### Data collection and processing

Gene expression profile data, and corresponding clinical data of 472 CM patients were retrieved from The Cancer Genome Atlas (TCGA) database for further analysis. GSE65904 (*N* = 210) and GSE19234 (*N* = 44) were obtained from GEO and used as independent verification datasets. A total of 1,316 EMT-related genes were downloaded from the molecular signature database v7.1 and the EMT gene database. When multiple probes were mapped to the same target gene, the average expression values of the probes were used to represent the final expression values of individual genes for the next analysis.

### Identification of ERGPs related to the prognosis of CM patients

To reduce the false discovery rate, we identified EMT-related genes with high variability before screening for prognostic-related ERGPs. EMT-related genes that met the screening criteria were then used to construct ERGPs. For example, when the expression value of the first EMT-related gene was greater than that of the second, the output score of this ERGP was “1” in a pair-wise comparison; otherwise, the output was “0.” Next, ERGPs with a score of 0 or 1 that accounted for less than 20 percent of the TCGA and GEO datasets were excluded. Univariate Cox proportional hazards regression analysis was used to determine ERGPs related to prognosis (*P* < 0.000005).

### Construction and evaluation of ERGP signature

To obtain the most optimized prognostic model, we adopted the least absolute shrinkage and selection operator (LASSO) method to further screen and obtain the ERGPs utilized to construct the model. Subsequently, the output scores of the prognostic ERGPs for each sample and the regression coefficients obtained by LASSO regression analysis were weighted, and a risk score formula was constructed. In TCGA dataset, a 5-year OS time-dependent ROC curve was utilized to determine the optimal cut-off value to distinguish between different risk groups. Survival curves were drawn using the Kaplan–Meier (K-M) method, and the log-rank test was used to evaluate the OS differences between the low- and high-risk groups. Time-dependent ROC curves and the area under the ROC curve (AUC) at 1-, 3- and 5-years were calculated to assess the sensitivity and specificity of the ERGP signature using the R package “timeROC.” Finally, we used univariate and multivariate Cox regression analyses to evaluate the prognostic value of ERGP signatures.

### Validation of the ERGPs signature

To verify the prognostic performance of the ERGPs signature, a separate external dataset, GSE65904 and GSE19234, was used for subsequent validation. The risk scores of all patients in the GEO cohort were calculated based on the risk scoring formula constructed in TCGA cohort, and all patients were divided into two groups using the same optimal cutoff values as those in TCGA cohort. The survival curves of the two groups of patients were drawn using the K-M method, and the log-rank test was used to determine the significance of the difference between the two groups. Finally, in the GSE65904 and GSE19234 cohorts, we integrated three clinical parameters and risk scores to verify the independent prognostic value of the ERGP signature.

### Assessment of the relationship between signature and clinicopathological parameters

We used the Wilcoxon rank sum test to analyze the correlation between clinicopathological parameters and risk scores in TCGA dataset. Statistical significance was set at *p* < 0.05. In addition, all patients were divided into different subgroups depending on their age, gender, stage, Breslow depth, and Clark level according to clinicopathological parameters to further evaluate the prognostic value of the ERGPs signature.

### Performance comparison of the ERGPs signature and existing prognostic models in survival prediction

To demonstrate the superior performance of the model developed in this study, we compared the ERGPs signature with two recently published gene signatures, a model consisting of five immune-related genes (Hu et al., 2020b) and a signature consisting of five IFNγ response-related genes (Hu et al., 2020a). The AUC was used to measure the predictive performance of each model.

### Functional enrichment analysis

To further explore the potential biological functions of EMT-related genes used to construct the ERGP signature, Gene Ontology (GO) and Kyoto Encyclopedia of Genes and Genomes (KEGG) enrichment analyses were performed. Metascape, an online tool with fast updation speed and comprehensive functions, was used for functional analysis.

### Assessment of tumor-infiltrating immune cells and immune checkpoints

Immune infiltration analysis was performed using the TIMER database, which permits to analyze the infiltration levels of six cells in 32 tumor types. The Spearman correlation coefficient test was used to evaluate the relationship between the infiltration levels of the six tumor immune infiltrating cells and the model’s risk score. As the effectiveness of immunotherapy and the expression levels of immune checkpoint genes were closely associated, we compared the differences in the expression levels of immune checkpoint genes between the high- and low-risk groups to screen for CM patients who would benefit from immunotherapy.

### Development and performance evaluation of nomogram

We integrated all the pathological parameters with independent prognostic significance in the multivariate Cox regression analysis results of TCGA dataset, and established a nomogram to predict the probability of OS in CM patients at 1, 3, and 5 years. A calibration curve was used to evaluate the performance of the nomogram. In addition, we utilized decision curve analysis (DCA) to evaluate the prediction accuracy of the nomogram.

### Statistical analysis

Except for the functional enrichment analysis, all statistical analyses were performed using the R software. The Spearman correlation test was used to calculate the correlation between the variables. A chi-square test was used to test for differences in the distribution of alive and dead between the high- and low-risk groups, and corrected *p*-values were used to determine whether there was statistical significance. Statistical significance was set at *p* < 0.05, unless otherwise stated.

## Results

### Establishment and evaluation of the ERGPs signature

**Figure 1:**
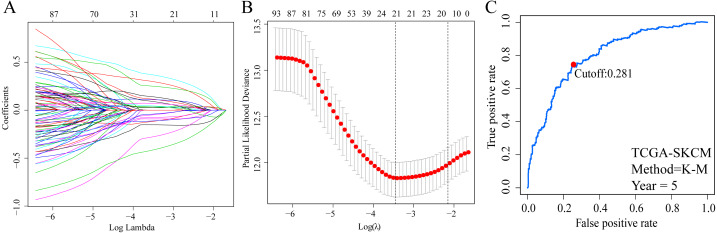
Screening of EMT-related gene pairs (ERGPs) used to construct prognostic signatures and determination of the best cut-off value of risk score. (A) Parameter filter by LASSO regress algorithm used five-fold cross-validation by through minimum criteria; (B) optimal feature selection based on LASSO coefficient profile plot of 21 ERGPs; (C) the optimal cut-off value of the ERGPs risk-score obtained by the time-dependent ROC curve analysis.

**Figure 2:**
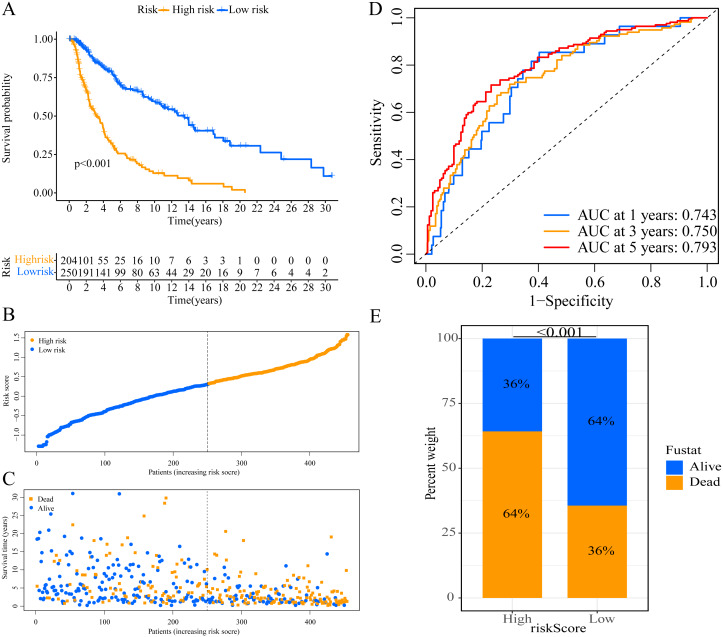
Establishment of overall survival (OS) prognostic model based on ERGPs. (A) Kaplan–Meier curves of OS according to ERGPs signature in the TCGA cohort; (B) distribution of patients’ risk scores; (C) patients’ survival time along with risk score; (D) time-dependent ROC curves of OS for the ERGPs signature score in the TCGA cohort at 1-, 3-, and 5 years; proportion of patients with different survival status in high and low risk groups.

**Figure 3:**
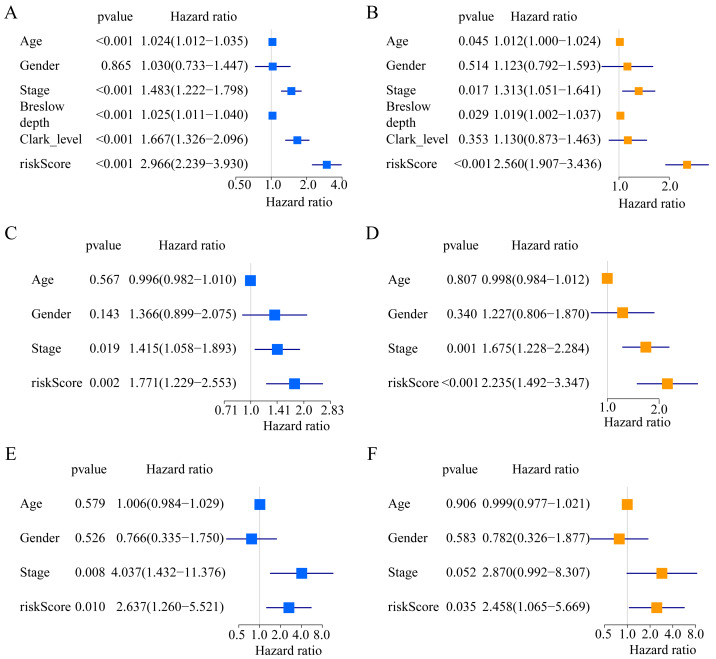
Forest plots of univariate and multivariate Cox regression analyses in different cohorts. (A, B) In the TCGA cohort. (C, D) in the GSE65904 cohort. (E, F) in the GSE19234 cohort.

A total of 373 EMT-related genes in the three datasets met the filter criteria of having a median absolute deviation >0.5; and 12,769 ERGPs were obtained for the subsequent construction of a new gene pair signature. In the discovery cohort (TCGA dataset), 104 ERGPs were significantly associated with prognosis in CM patients according to univariate Cox regression analysis. Among these, 21 ERGPs were further selected using Lasso-Cox regression analysis to establish a prognostic risk signature (Figs. 1A–1B). The selected 21 ERGPs comprised 33 EMT-related genes (Table 1). Time-dependent ROC curve analysis indicated that the optimal truncation value of the risk score was 0.281 (Fig. 1C). Based on this cutoff value, all CM patients were divided into two groups with different survival outcomes. The OS, disease-specific survival, and progression-free survival of CM patients in the high-risk group were shorter (*P* < 0.001, Fig. 2A, Figs. S1A–S1D). In addition, increasing risk scores were associated with a higher mortality (Figs. 2B–2C). In TCGA cohort, the AUC values of the model were 0.743, 0.750, and 0.793 for predicting 1-year, 3-year, and 5-year survival rates, respectively (Fig. 2D). Mortality was significantly higher in the high-risk group (*P* < 0.001, Fig. 2E). In addition, univariate and multivariate Cox regression analyses showed that risk scores independently predicted outcomes in patients with CM (HR, 2.560; 95% CI [1.907–3.436]; *P* < 0.001, Figs. 3A, 3B).

**Table 1:** Prognostic signature consists of 21 EMT-related gene pairs.

Signature		Gene A		Gene B		Coefficient
pair1		AFAP1L2		MMP11		−0.114
pair2		BCL6		NOTCH3		−0.111
pair3		BIRC5		WIPF1		0.364
pair4		CAP2		LYN		0.111
pair5		CAP2		PTPN6		0.108
pair6		CCL5		THY1		−0.091
pair7		CDH1		CXCL9		0.034
pair8		CXCL13		SOX9		−0.076
pair9		CXCL9		LUM		−0.153
pair10		ECM1		ISG15		0.294
pair11		FBP1		NOTCH3		−0.046
pair12		GAB2		WIPF1		0.025
pair13		JUN		KIT		−0.273
pair14		KIT		PDGFD		0.248
pair15		KLF5		PSTPIP1		0.087
pair16		NMI		PDGFRB		−0.41
pair17		NOTCH3		SNTB1		0.043
pair18		PDGFRB		SOD2		0.069
pair19		PDGFRB		ST6GAL1		0.137
pair20		RUNX3		WIPF1		0.0323
pair21		TNFSF10		VCAN		−0.039

### Verification of the ERGPs signature in GEO database

**Figure 4:**
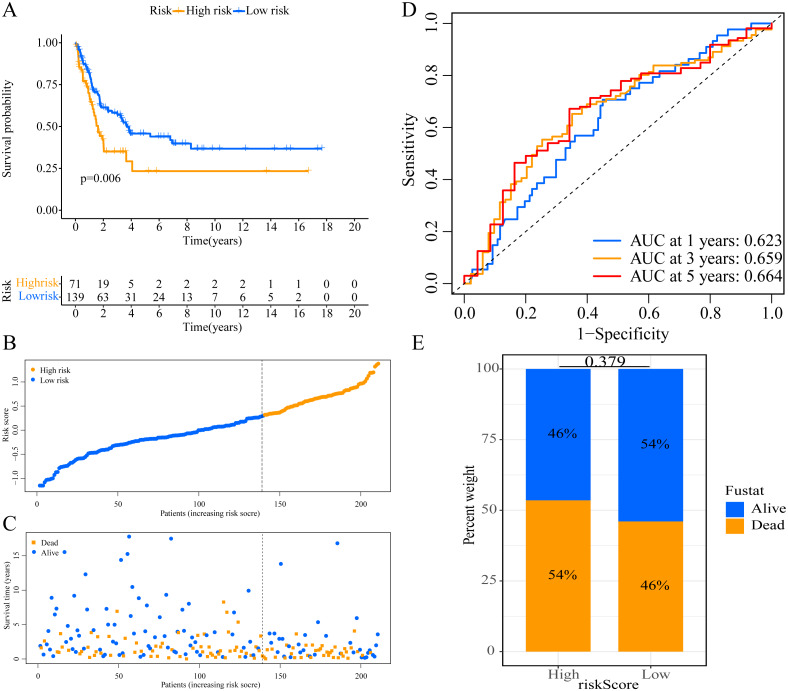
External validation of the prognostic model in the GSE65904 cohort. (A) Kaplan–Meier curves of OS according to ERGPs signature; (B) distribution of patients’ risk scores; (C) patients’ survival time along with risk score; (D) time-dependent ROC curves of OS for the ERGPs signature score at 1-, 3-, and 5 years; proportion of patients with different survival status in high and low risk groups.

**Figure 5:**
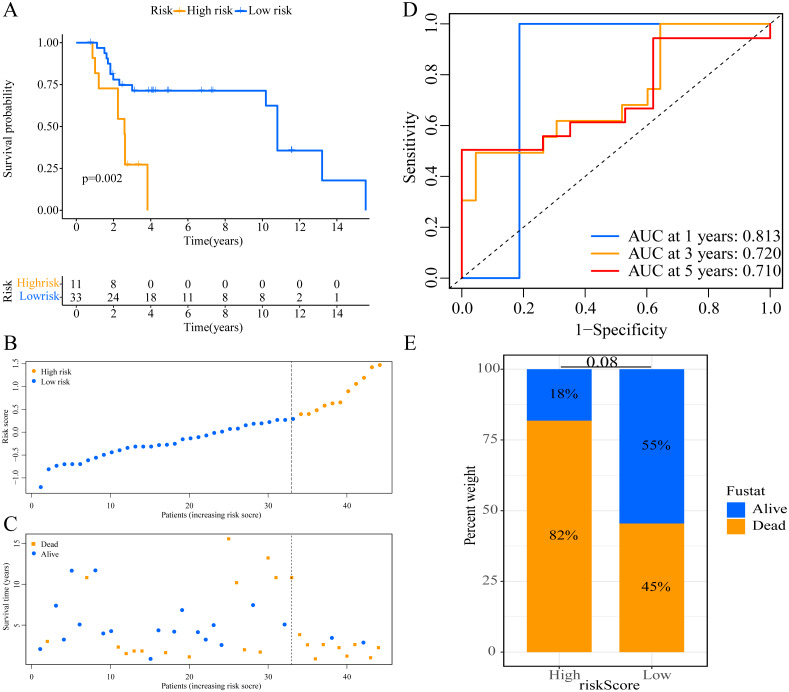
External validation of the prognostic model in the GSE19234 cohort. (A) Kaplan–Meier curves of OS according to ERGPs signature; (B) distribution of patients’ risk scores; (C) patients’ survival time along with risk score; (D) time-dependent ROC curves of OS for the ERGPs signature score at 1-, 3-, and 5 years; (E) proportion of patients with different survival status in high and low risk groups.

We used the risk score formula constructed in TCGA cohort to calculate the risk score of each patient in the GSE65904 and GSE19234 datasets, and divided all patients into low- and high-risk groups using the previously indicated cutoff value. As expected, K-M analysis indicated that patients in the high-risk group had worse OS than those in the low-risk group (GSE65904: *P* = 0.006, Fig. 4A; GSE19234: *P* = 0.002, Fig. 5A). The risks score and survival status distribution in the GSE65904 and GSE19234 datasets are shown in Figs. 4B, 4C, 5B and 5C, respectively. Next, we calculated AUC values to assess the predictive performance of the model in the GSE65904 cohort. The AUC values were 0.623, 0.659, and 0.664 for 1-year, 3-year, and 5-year survival, respectively (Fig. 4D). The mortality in the high-risk group (54% of the total) was higher than that in the low-risk group, but the difference between the two groups was not statistically significant (*P* = 0.379; Fig. 4E). In the GSE19234 dataset, the AUC of 1-year survival was 0.813, the AUC of 3-year survival was 0.720, and the AUC of 5-year survival was 0.710 (Fig. 5B). Similar to the results of the GSE65904 cohort, the mortality in the high-risk group was higher than that in the low-risk group, but the difference was not statistically significant (*P* = 0.08; Fig. 5E). The independent prognostic analysis indicated that the risk score was an independent prognostic parameter in both datasets, regardless of the other clinicopathological characteristics of CM patients (for GSE65904: HR, 2.235; 95% CI [1.492–3.347]; *P* < 0.001, Figs. 3C, 3D; for GSE19234: HR = 2.458, 95% CI [1.065–5.669], *P* = 0.035, Figs. 3E, 3F). The above results fully validate the prognostic value of the developed model.

### Association between the signature risk score and clinicopathological parameters

**Figure 6:**
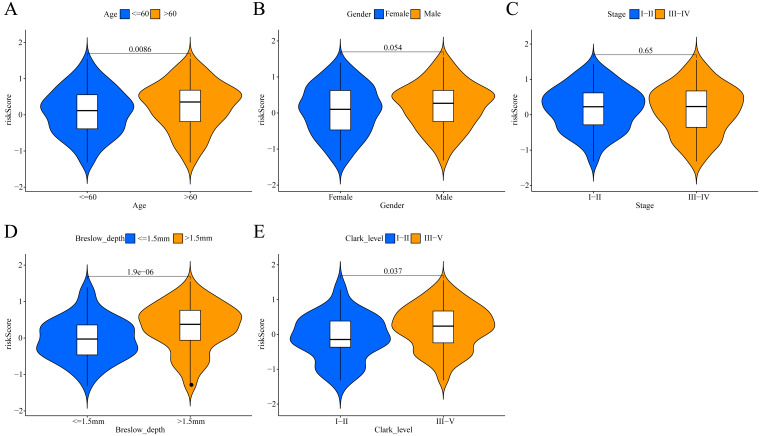
Association between the ERGPs signature risk score and clinical parameters in the TCGA cohort. (A) Age (≤60 and >60); (B) gender (female and male ); (C) stage (I–II and III–IV); (D) breslow_depth (≤1.5 and >1.5); (E) clark_level (I–II and III–IV).

To investigate the potential clinical value of the risk scores determined by the ERGPs signature, a Wilcoxon rank sum test was performed to assess whether they correlated with clinicopathological parameters. In TCGA cohort, the risk scores were positively correlated with age, Breslow depth, and Clark level, as the risks scores were significantly higher in the groups with age >60, Breslow depth >1.5 mm, and Clark level III-IV (Figs. 6A, 6D, 6E). However, there was no significant association between the risk score and sex or tumor stage (Figs. 6B, 6C).

### Comparison of the performance of the ERGPs signature with the other two models

**Figure 7:**
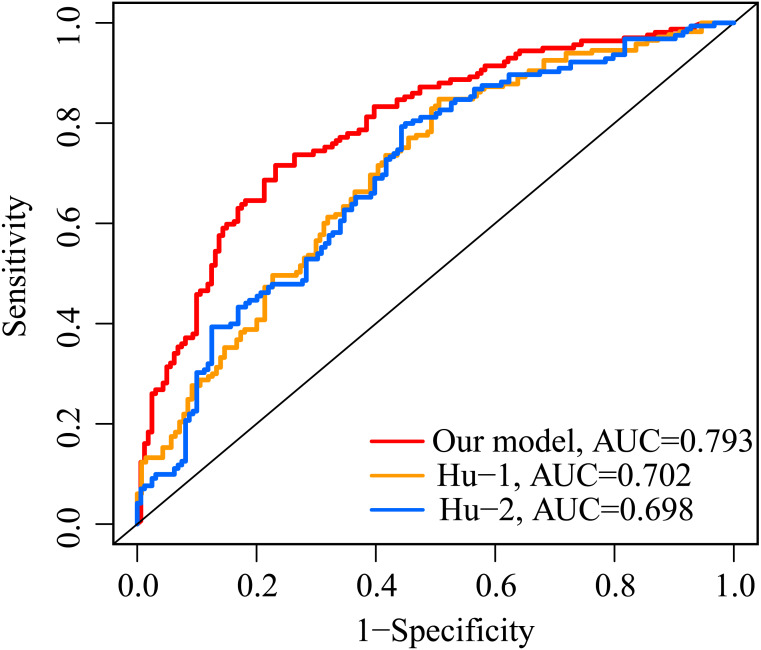
Comparison of ROC analysis between our model and two other existing models in predicting 5-year overall survival. Red: the model constructed for this study. Yellow: IFNγ response-related signature.Blue: immunogenomic profiling identifies prognostic signature.

The AUC value of our ERGP signature for predicting 5-year OS was 0.793 (Fig. 7). The AUC values of the IFNγ response-related model and the immune-related gene model established by Hu et al. in predicting 5-year OS were 0.702 and 0.698, respectively (Fig. 7). This highlighted the superior performance of our newly established ERGPs signature.

### Functional enrichment analysis

**Figure 8:**
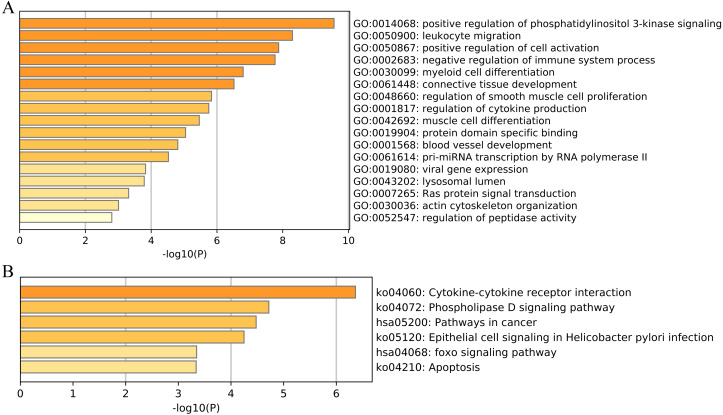
Functional enrichment of 33 unique EMT-related genes using the Metascape database. (A) GO enrichment analysis; (B) KEGG enrichment analysis.

We then performed functional and pathway enrichment analyses of 33 unique EMT-related genes that were used to construct the ERGPs signature. GO enrichment analysis indicated that these genes were involved in many important biological processes, including positive regulation of cell activation, blood vessel development, and positive regulation of phosphatidylinositol 3-kinase signaling (Fig. 8A). KEGG analysis showed that these genes were mainly enriched in pathways related to cytokine-cytokine receptor interaction, cancer, and FOXO signaling (Fig. 8B). This indicated that these genes are involved in cancer regulation, suggesting they may aid in the prediction of the prognosis of patients with CM.

### Analysis of tumor-infiltrating immune cells

**Figure 9:**
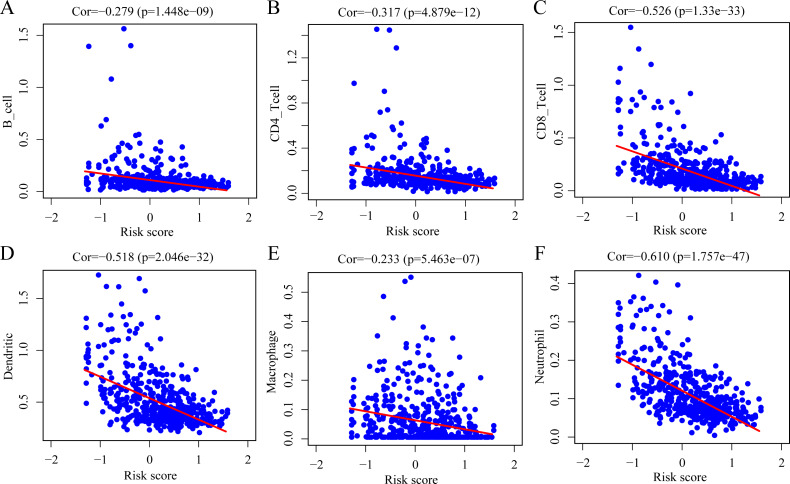
Spearman correlation of the risk score and infiltration abundance of six types of immune cells. (A) B cell; (B) CD4 T cell; (C) CD8 T cell; (D) dendritic cell; (E) macrophage cell; (F) neutrophil cell.

To determine whether the ERGPs signature can effectively reflect the tumor immune microenvironment status, we evaluated the correlation between the risk score and the immune cell infiltration levels. As shown in Fig. 9, there was a negative correlation between the risk score and the abundance of all six immune infiltrating cells, including B cells (r = −0.279), CD4 T cells (r = −0.317), CD8 T cells (r = −0.526), dendritic cells (r = −0.518), macrophages (r = −0.233), and neutrophils (r = −0.610).

### Analysis of expression levels of immune checkpoint molecules

**Figure 10:**
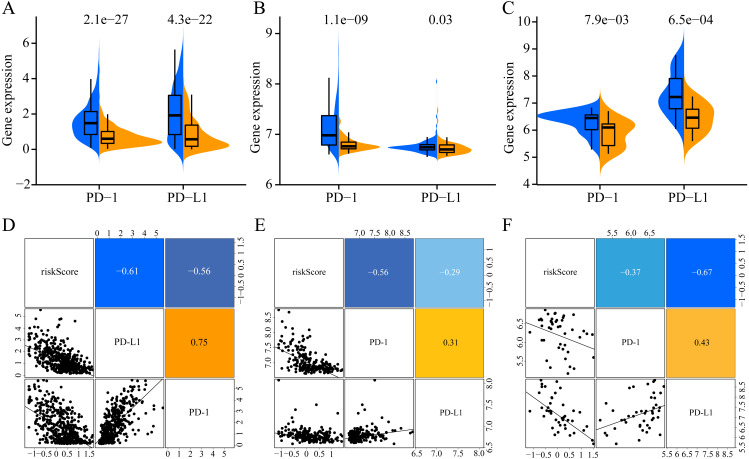
Association of ERGPs characteristics with immune checkpoint genes. Expression of PD-1 and PD-L1 between high and low risk groups in the three datasets TCGA (A), GSE65904 (B) and GSE19234 (C). Correlation between risk score and PD-1 and PD-L1 expression levels in three TCGA (A), GSE65904 (B) and GSE19234 (C) datasets.

To increase the clinical utility of the prognostic model, we further investigated the association between the risk score and expression levels of the genes encoding the checkpoint proteins programmed death-1 (PD-1) and programmed death ligand 1 (PD-L1) in the three datasets. The low-risk groups in the three cohorts presented higher expression of PD1 and PD-L1 than that in the high-risk group (Figs. 10A–10C). In addition, the expression levels of PD-1 and PD-L1 were negatively correlated with the risk score(for TCGA: PD-1 R = −0.56, PD- L1 R = −0.61, Fig. 10D; for GSE65904: PD-1 R = −0.56, PD-L1 R = −0.29, Fig. 10E; for GSE19234: PD-1 R = −0.37, PD-L1 R = −0.67, Fig. 10D). Altogether, these results indicate that CM patients in the low-risk group may benefit more than those in the high-risk group from immunotherapy.

### Construction and evaluation of the nomogram

**Figure 11:**
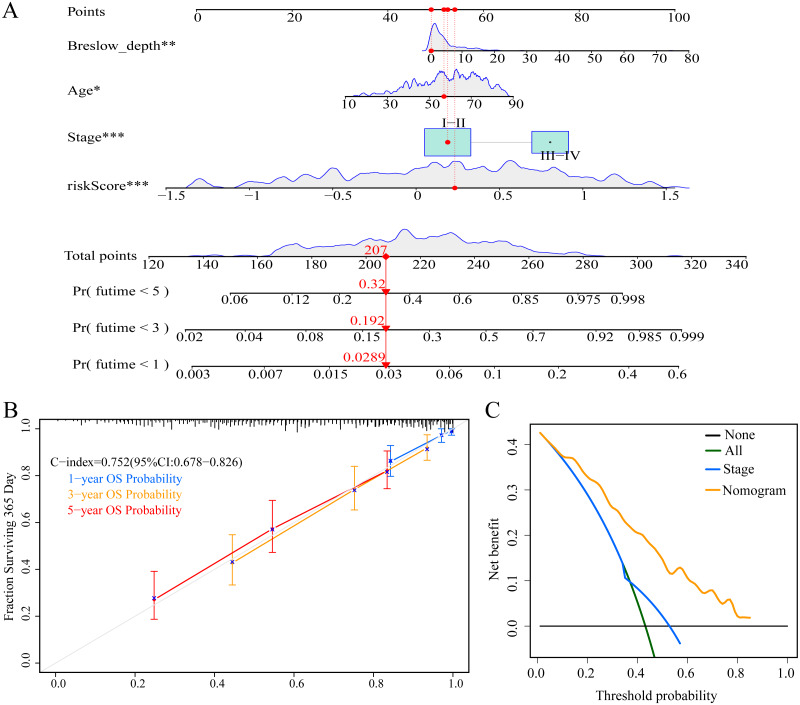
Construction and evaluation of nomogram. (A) Nomogram predicting overall survival probability for patients with CM in the TCGA cohort; (B) calibration curves and C index of the nomogram; (C) decision curve analyses comparing nomogram and AJCC stage in TCGA cohort.

To enable individualized prediction of the prognosis of CM patients, we integrated all clinicopathological parameters with independent prognostic significance from TCGA dataset as well as risk scores into the novel nomogram (Fig. 11A). The calibration curves of the 1-year, 3-year, and 5-year OS rates showed good consistency between the predicted value and the actual observed value of the nomogram (Fig. 11B). The C-index of the nomogram prediction performance was 0.752 (95% CI [0.678–0.826]) (Fig. 11B). Furthermore, DCA agreed with the results of our study that this novel nomogram had a better net benefit than that of traditional American Joint Committee on Cancer (AJCC) stage (Fig. 11C).

## Discussion

CM is a malignant tumor with poor prognosis, characterized by strong aggressiveness and poor response to radiotherapy and chemotherapy (Miller & Mihm Jr, 2006; Terando, Sabel & Sondak, 2003). Therefore, the construction of a robust and accurate prognostic signature is particularly important for improving the prognosis of patients with CM. Despite previously published signatures based on mRNA and lncRNA that can predict the prognostic outcome of CM patients (Chen et al., 2017; Han et al., 2020), these signatures have not been incorporated to a clinical setting due to the inherent heterogeneity of tumors and the difficulty of data standardization caused by cross-platform sequencing. In this study, we successfully developed a signature consisting of 21 ERGPs and verified its performance in two independent GEO datasets. The signature could effectively divide CM patients into two groups with different OS, with the higher risk group having a lower survival rate. Further analysis of the clinicopathological parameters and risk scores in TCGA dataset showed that the risk score was an independent prognostic factor, which was verified in the GSE65904 and GSE19234 cohorts. In addition, the prognostic models of ERGPs in the TCGA, GSE65904, and GSE19234 datasets had higher AUC values than those of two previously established prognostic models. In addition, there were significant differences in the expression of immune checkpoint genes (PD-1 and PD-L1) between the high-and low-risk groups, which indicated that they could be related to the prognosis of patients and new therapeutic targets. Finally, the nomogram constructed by combining the risk scores and clinicopathological parameters could accurately predict the survival rate of CM patients, and had a better performance than that of the AJCC stage model.

As the role of EMT-related genes in tumor development is widely known, and gene pair signatures are a novel and reliable algorithm that can overcome multiple drawbacks; we used ERGPs to construct a prognostic signature. Despite the large amount of publicly available data permits to construct accurate gene signatures, use of this data is required to overcome the significant technical challenges posed by data diversity. Because of the biological heterogeneity between different datasets and the technical noise caused by cross-platform sequencing, algorithms that use gene expression profiles require the use of normalized data, which is a challenging task (Leek et al., 2010). However, the novel algorithm used in this study does not require preprocessed data, and it has yielded reliable results in a variety of previous studies (Du & Gao, 2020; Hong et al., 2020; Huo, Wu & Zang, 2020; Kang, Jia & Liu, 2020). Many of the 33 unique EMT-related genes that construct the ERGP signature have been studied in several tumors, including melanoma. For example, it is known that CAP2 plays an important role in the invasion of malignant melanoma, and that its high expression is associated with a poor prognosis in patients with malignant melanoma (Masugi et al., 2015). Previous studies have shown that RUNX3 can independently predict the prognosis of patients with melanoma, and that patients with positive RUNX3 expression have a better 5-year survival rate (Zhang et al., 2011). GAB2 was expressed at higher levels in metastatic melanoma than in primary melanoma, and it could enhance the invasiveness of melanoma cells by inhibiting the PI3K-Akt pathway (Horst et al., 2009). ECM1 regulates breast cancer cell invasion by inducing the expression of genes associated with the progression of EMT (Lee et al., 2015). Other studies have shown overexpression of ISG15 at either the mRNA or the protein level, which was related to the poor prognosis of breast cancer patients (Kariri et al., 2020). Despite the fact that a remarkable proportion of the genes included in our signature had been previously related to cancer, a relationship is still unknown for some other of the genes, suggesting this should be further explored in the future.

It is well known that there is a strong link between tumor-infiltrating immune cells and patient prognosis (Mahmoud et al., 2012; Pagès et al., 2005; Pagès et al., 2010). Therefore, we used the Spearman correlation coefficient to evaluate the association between the risk score and the abundance of six types of tumor-infiltrating immune cells. As the risk score increased, the levels of tumor-infiltrating immune cells decreased. This suggests that the established ERGP signature could reflect the immune microenvironment status of CM patients to a certain extent. Previous studies have investigated the relationship between B cells, dendritic cells, and neutrophils and the prognosis of CM patients, and reported that patients with reduced infiltration levels of these immune cells had a poorer prognosis, which was consistent with our findings (Gross et al., 2017; Iglesia et al., 2016; Selitsky et al., 2019). The results of this study may help to elucidate the reason underlying the poorer prognosis of CM patients in the high-risk group.

In recent years, immune checkpoint inhibitors targeting PD1 and PD-L1 have been successfully used as a treatment strategy for many malignant tumors (Herbst et al., 2016; Topalian et al., 2012). Unfortunately, only a small percentage of patients benefit from it (Darvin et al., 2018), highlighting the need to identify biomarkers that can predict which patients will benefit from this therapy in advance. Our results suggest a significant association between the ERGP signature and the expression levels of PD-1 and PD-L1. In addition, PD-L1 can be used a biomarker for predicting the response to immunotherapy using immune checkpoint inhibitors (Havel, Chowell & Chan, 2019). Since the expression levels of PD-1 and PD-L1 are negatively correlated with risk scores, CM patients in the low-risk group may benefit more from immunotherapy than those in the high-risk group. Therefore, we believe that our ERGP signature can be used to guide clinicians’ choice on the most suitable treatment strategy.

Nomograms are intuitive tools that can predict the survival probability of an individual patient and have been widely used in previous studies (Balachandran et al., 2015; Harrell Jr, Lee & Mark, 1996). To our knowledge, this is the first nomogram constructed based on the signature of ERGPs combined with age, stage, and Breslow depth. Both the C index and calibration chart showed that our nomogram had excellent performance. Remarkably, the DCA showed that the net benefit of the nomogram based on the ERGP signature was higher than that of the currently used AJCC staging system. Altogether, these results indicate that our nomogram can be a reliable tool for developing individualized treatment plans for patients, thus improving their prognosis.

Our study has several strengths. First, the novel algorithm we used does not require preprocessing of the cross-platform data when constructing a prognostic model. Second, the optimal cutoff value for the risk score can be applied to any of the external datasets. Third, our prognostic model performed better than the two previously established predictive models. Finally, the nomogram predicted the survival probability of a single patient more accurately than the traditional AJCC staging system. However, some limitations must be considered. This is a retrospective study based on a publicly available database, and these results need to be verified in a prospective cohort in future studies. In addition, our study did not validate 33 EMT-related genes *in vitro* or *in vivo*, which remains a task for future studies.

## Conclusion

In summary, our study used a novel algorithm to develop a reliable signature of ERGPs that accurately distinguishes CM patients into a high-risk group with a poor prognosis and a low-risk group with a better prognosis. The ERGP signature was also able to independently and accurately predict the prognosis of CM patients in two independent external datasets (GSE65904 and GSE19234). In addition, our model predicted the greatest benefit for CM patients in the low-risk group when treated with PD-1 and PD-L1 inhibitors. These results suggest that our signature can help in the personalized treatment of patients with CM.

## Supplemental Information

10.7717/peerj.12646/supp-1 Supplemental Information 1 Establishment of disease-specific survival and progression-free survival prognostic model based on ERGPs (A) Kaplan–Meier curves of disease-specific survival according to ERGPs signature in the TCGA cohort; (B) Time-dependent ROC curves of disease-specific survival for the ERGPs signature score in the TCGA cohort at 1-, 3-, and 5 years; (C) Kaplan–Meier curves of progression-free survival according to ERGPs signature in the TCGA cohort; (D) Time-dependent ROC curves of progression-free survival for the ERGPs signature score in the TCGA cohort at 1-, 3-, and 5 years.

## Additional Information and Declarations

### Data Availability

The following information was supplied regarding data availability: Data is available at NCBI GEO: GSE65904.
