# Supplementary figures and images for: Identification and validation of an epithelial mesenchymal transition-related gene pairs signature for prediction of overall survival in patients with skin cutaneous melanoma
Source: PeerJ. 2022 Jan 21;10:e12646. doi: 10.7717/peerj.12646 (PMC8785661; doi:10.7717/peerj.12646)

A

Risk — High risk — Low risk

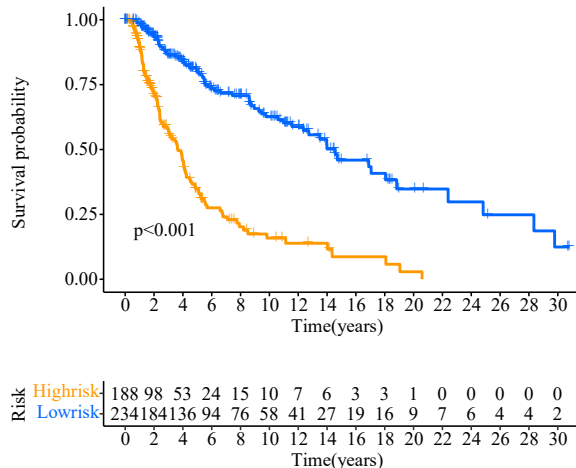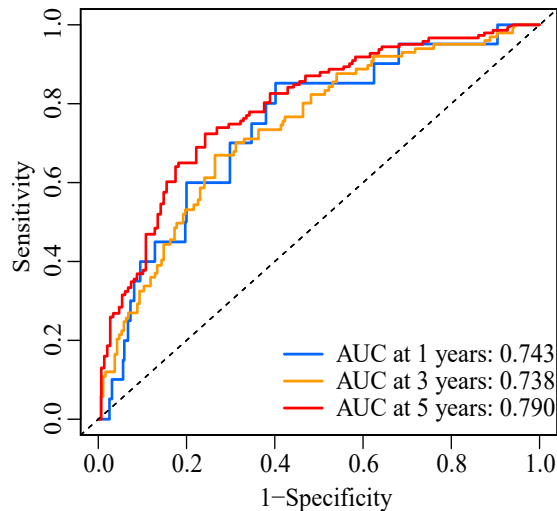

B

Risk — High risk — Low risk

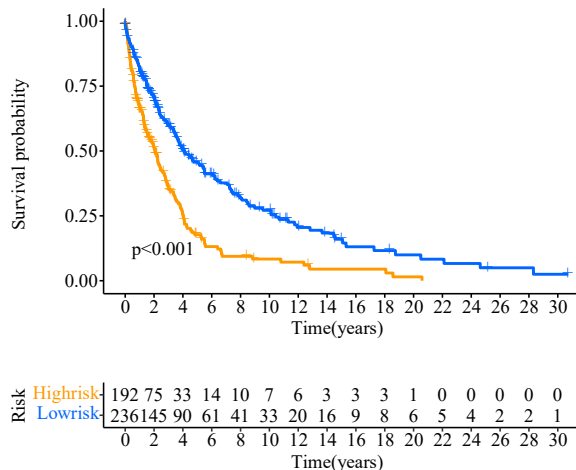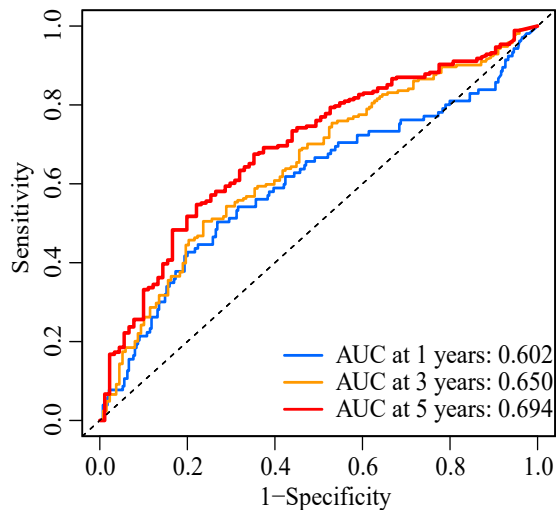

Supplement: Supplemental Information 1 — (A) Kaplan–Meier curves of disease-specific survival according to ERGPs signature in the TCGA cohort; (B) Time-dependent ROC curves of disease-specific survival for the ERGPs signature score in the TCGA cohort at 1-, 3-, and 5 years; (C) Kaplan–Meier curves of progression-free survival according to ERGPs signature in the TCGA cohort; (D) Time-dependent ROC curves of progression-free survival for the ERGPs signature score in the TCGA cohort at 1-, 3-, and 5 years. [file peerj-10-12646-s001.pdf]
